# In vitro preliminary study on different anti-PD-1 antibody concentrations on T cells activation

**DOI:** 10.1038/s41598-022-12136-1

**Published:** 2022-05-19

**Authors:** Irena Wieleba, Kamila Wojas-Krawczyk, Izabela Chmielewska, Magdalena Wójcik-Superczyńska, Paweł Krawczyk, Janusz Milanowski

**Affiliations:** Pneumonology, Oncology and Allergology Department, Medical University of Lublin, Jaczewskiego 8, 20-954 Lublin, Poland

**Keywords:** Cancer, Immunology, Oncology

## Abstract

Lung adenocarcinoma predominates among diagnosed nonsmall cell lung cancer subtypes in nonsmokers. The introduction of immune checkpoint inhibitors into clinical practice offered patients prolonged progression-free survival and overall survival times. However, the results demonstrate that the benefits do not apply to all patients. Nivolumab is a monoclonal antibody against the PD-1 protein expressed mainly on T lymphocytes and is widely used in cancer therapy in different settings. Tumor cells often express the PD-L1 molecule and can effectively block the action of PD-1-positive lymphocytes. A body of knowledge regarding the high expression of PD-L1 on tumor cells highlights that it does not always correlate with the effectiveness of anti-PD-1 therapy. The side effects of the therapy also constitute a significant issue. These side effects can occur at any time during anti-PD-1 treatment and lead to discontinuation and even the death of the patient. In these situations, it is possible to delay the dosage. Nevertheless, unfortunately, it is not possible to reduce the dose of anti-PD-1 antibody, which would undoubtedly minimize side effects, leaving the patient's immune system active. In our preliminary study, we analyzed the effect of different concentrations of nivolumab on the functioning of T lymphocytes. Activation and proliferation markers were investigated on T cells after being cultured with antigen-stimulated autologous dendritic cells. This process may indicate an appropriate concentration of nivolumab, which shows clinical activity with minimal side effects.

## Introduction

Lung adenocarcinoma is one of the most common types of nonsmall cell lung cancer in nonsmokers^1^. Immunotherapy with immunological checkpoint inhibitors (ICIs) has revolutionized cancer treatment, especially for patients without actionable driver mutations. Two groups of ICIs are widely used in diverse cancer treatments and different line settings. The first group consists of anti-PD-1 (programmed death 1) antibodies, which include pembrolizumab and nivolumab. Both monoclonal antibodies block the programmed death 1 receptor on the lymphocyte surface, resulting in increased activity of these cells. The second group of ICIs includes anti-PD-L1 antibodies, including atezolizumab, durvalumab, and avelumab, which block the ligand for PD-1–PD-L1—on tumor cells and tumor-infiltrating immune cells^2,3^.

It is well known that the PD-1/PD-L1 pathway is a mechanism that protects our body against overactivation of the immune system and directs its activity against healthy tissues. More critically, tumor cells, by expressing the PD-L1 molecule on their surface, could very effectively suppress the activity of the immune system^4,5^. Therefore, blocking these molecules with specific antibodies has become a very effective form of cancer treatment. One should remember that in addition to the proper qualification of patients for immunotherapy based on the registration rules, the effectiveness of ICIs depends on the conditions of the patient's immune systems. Adequate functioning of the anticancer immune system requires the appropriate interaction of many elements of specific and nonspecific responses. Consequently, the pre-existing immunity in the tumor site and the preservation of an active immune system in the peripheral blood determine the survival of immunized patients and the chances of responding to immunotherapy^6^.

A necessary condition for the safe inclusion of ICIs is the elimination of contraindications, predominantly active or past autoimmune diseases. The inclusion of ICIs in those patients could result in a severe exacerbation of the autoimmune process, a potential threat to the patient's life. Adverse events of immunotherapy result from overstimulation of the immune system, which triggers inflammatory responses. Unfortunately, in the registration rules of immunotherapeutic agents, there is no option to reduce the drug dose when adverse events occur. The proposed rule in immunotherapy would be adjusting the safe immunotherapy dose for the patient while blocking cancer cells and stimulating the immune system.

In the present preliminary study, we examined the effect of different nivolumab concentrations on T cell activity in in vitro cultures. We proved that the generation of fully functional dendritic cells from lung cancer patients possibly indicates a lack of functional exhaustion of the immune system in the peripheral blood. This study may indicate at which doses of anti-PD-1 antibody stimulate the immune system, which may minimize the side effects of such therapies in the future.

## Results

### Immunophenotype of generated dendritic cells

**Figure 1:**
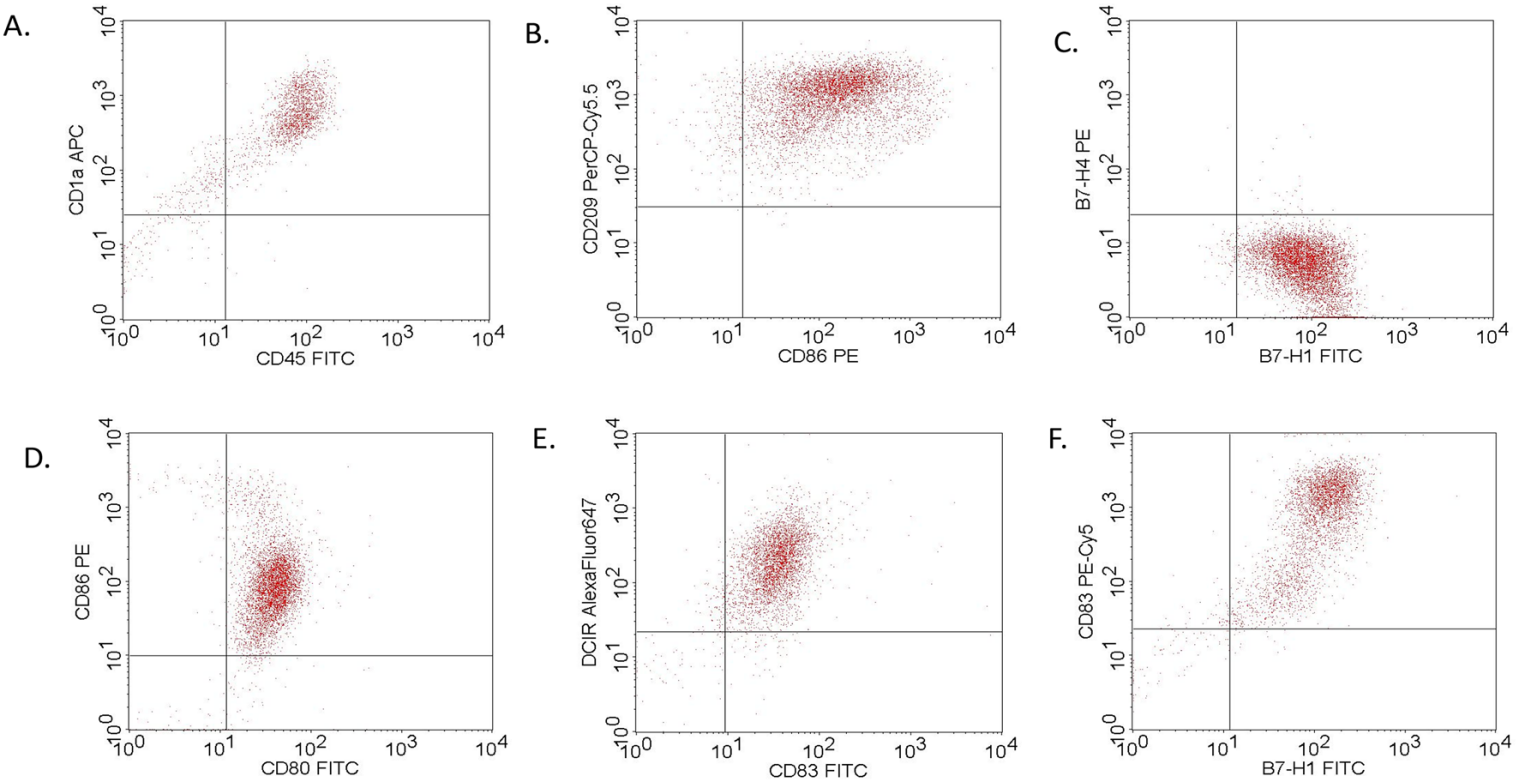
Dendritic cell phenotype analysis by flow cytometry: (**A**) generated DCs expressed CD1a and CD45; (**B**) expression of CD209 and CD86 was observed; (**C**) no expression of B7-H4 was observed, and only B7-H1 was expressed by generated DCs; (**D**) DCs expressed CD80/86 markers; (**E**) generated DCs expressed DCIR molecules; F. DCs expressed both CD83 and B7-H1 markers.

Generated dendritic cells expressed the following markers: CD1a, CD11c, CD80/86, CD83, CD209, B7DC, and B7-H1. Lake or low percentages of cells expressing B7-H4 markers were observed. Thus, we obtained the pull of mature dendritic cells with the ability of antigen presentation for lymphocytes. PD-L1 and PD-L2 expression was a desirable effect in our analysis of nivolumab effectiveness. Representative dot plots from cytometry analysis are presented in Fig. 1.

### Effect of different concentrations of nivolumab on the T cell immunophenotype

*1. Assessment of the percentage of T helper and T cytotoxic cells with the expression of the activation markers CD25, CD69, and CD95*

In the T cytotoxic (CD8-positive) cell subpopulation, a significantly higher percentage of CD25-positive cells was observed in all tested concentrations of nivolumab compared with the control culture. The highest percentage of cells expressing the CD25 marker was detected in the probe treated with 10 μg/ml nivolumab, which was approximately twice as high as that in the control culture (*p* < *0.05*). Similarly, the highest percentage of CD69-positive cells was observed for 10 μg/ml nivolumab. The results obtained for the remaining probes were similar and approximately 2.5 × times higher than in the nontreated control probe. The percentage of cells expressing the CD95 marker was slightly higher in all treated probes *vs*. the control. Thus, the addition of different concentrations of nivolumab appears to affect early cell activation directly and induce late-activity markers to a minor extent.

**Figure 2:**
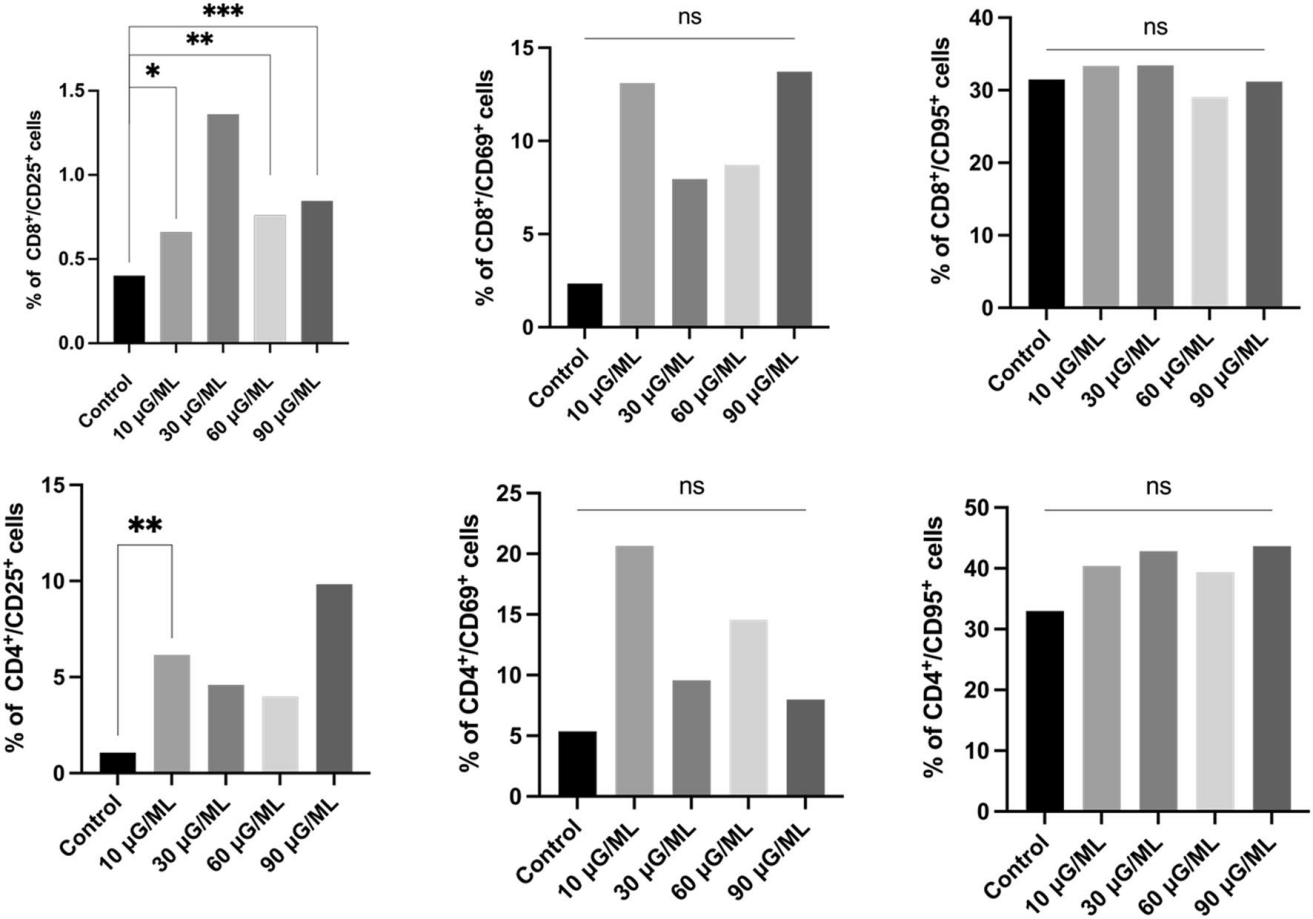
Representative bolt graph from the analysis of cell percentage with an expression of CD25, CD69 and CD95 markers in a subpopulation of Tc and Th cells' *, **, ***—describe statistically significant differences from Wilcoxon matched pair test, p-value < 0.05 (individual p-value for each group are described in the Table 1); ns—nonsignificant.

Similar to CD8-positive lymphocytes, within CD4-positive T cells, we observed a significant increase in the percentage of double-positive (CD4^+^/CD25^+^) cells after nivolumab treatment compared with the nontreated control. Moreover, higher percentages of CD4^+/^CD69^+^ and CD4^+^/CD95^+^ cells were observed in all tested concentrations of nivolumab vs. control culture, but the data were not statistically significant. The highest percentage of cells expressing CD25, CD69 and CD95 markers was observed for probes treated with 10 μg/ml nivolumab. Thus, it seems that nivolumab, even at minimal concentrations, impacted T helper cells and could induce the expression of activation markers. The representative graph analysis is shown in Fig. 2.

*2. Assessment of the percentage of Th and Tc cells expressing intracellular 107a marker*

**Figure 3:**
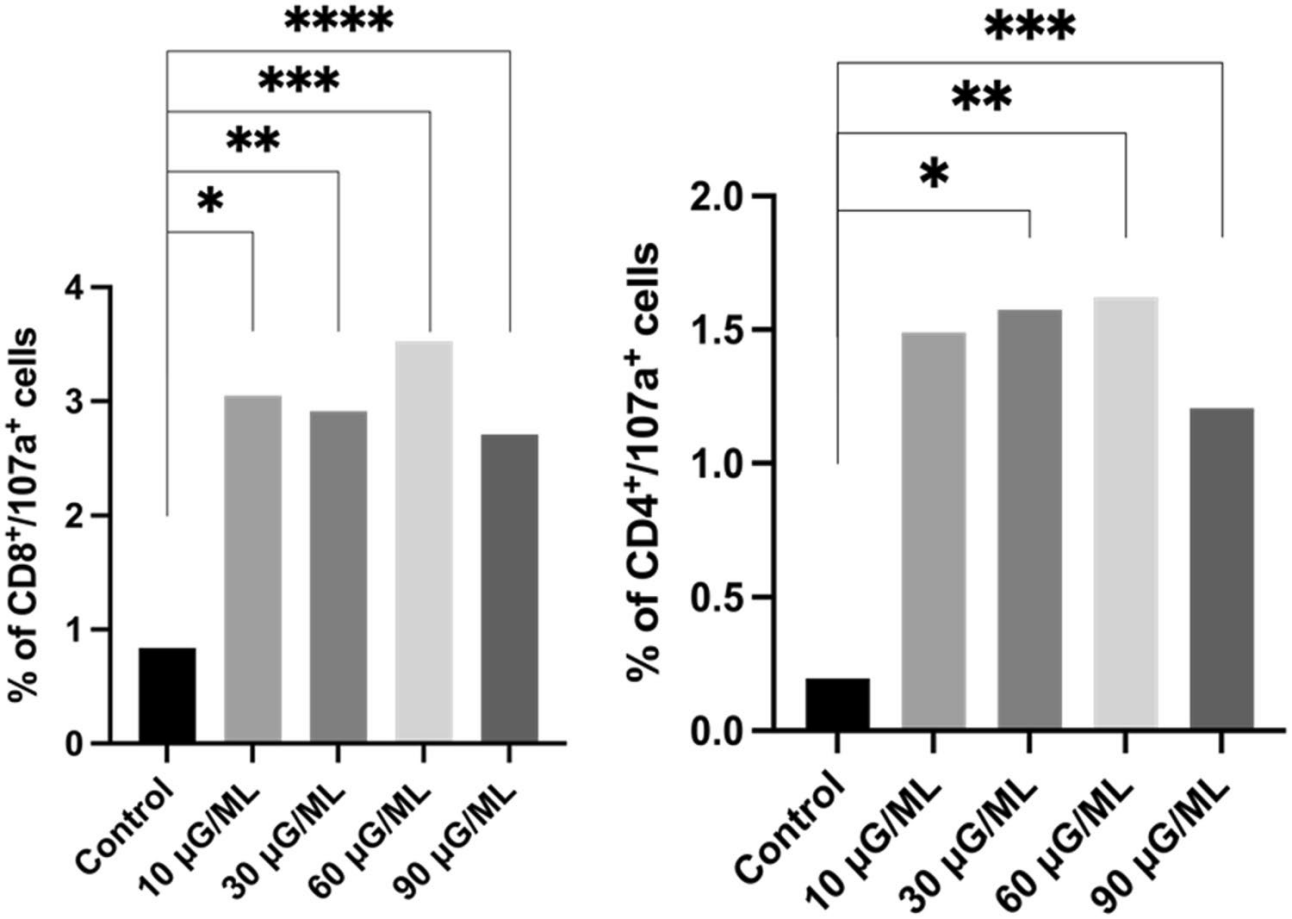
Representative bolt graph from the analysis of cell percentage with an expression of intracellular 107a marker in a subpopulation of Tc and Th cells' *, **, ***,****—describe statistically significant differences from Wilcoxon matched pair test, p-value < 0.05 (individual p-value for each group are described in Table 1).

CD107a has been described as a marker of cytotoxic CD8-positive cell degranulation, and its expression indicates the cytolytic activity of cells. We observed a significant increase in the percentage of cells expressing the marker 107a in both T cell-CD4- and CD8-positive subpopulations. The representative graphs' analysis is shown in Fig. 3.

*3. Assessment of the percentage of T helper cells expressing receptors for interleukin 4 (CD124) and interleukin 12 (CD212)*

**Figure 4:**
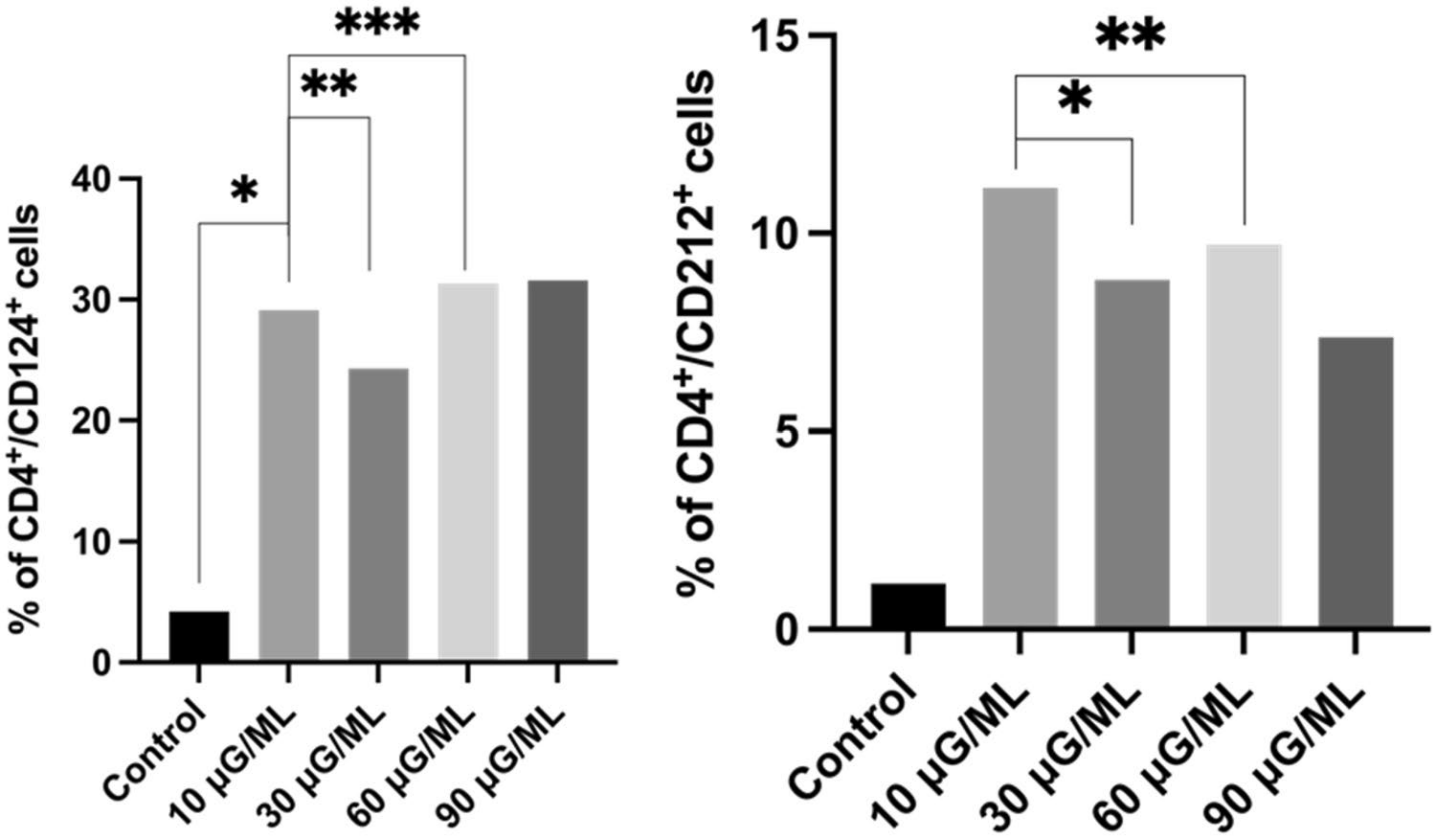
Representative bolt graph from the analysis of cell percentage with an expression of CD124 and CD212 markers in subpopulation Th cells;' *,**,***—describe statistically significant differences from Wilcoxon matched pair test, p-value < 0.05 (individual p-value for each group are described in Table 1), ns—nonsignificant.

We determined a higher percentage of Th cells expressing CD124 and CD212 for all tested concentrations of nivolumab when compared with the control culture. The percentage of T helper cells expressing the IL-4 receptor (CD124) was significantly higher after a 10 µg/ml dose of nivolumab than after unstimulated culture (p < 0.05). A nonsignificant increase in the percentage of T helper cells expressing IL-4 receptor (CD124) was observed when further increasing the nivolumab dose, and it remained constant at concentrations of 60 µg/ml and 90 µg/ml. On the other hand, a significant increase in the number of T helper cells expressing the IL-12 receptor was observed after 30 µg/ml nivolumab compared to 10 µg/ml nivolumab (p = 0.0208) and in the 60 µg/ml stimulated culture compared to the 10 µg/ml culture (p = 0.0218). The representative graphs' analysis is shown in Fig. 4.

*4. Assessment of the Th and Tc subpopulations expressing CD28*

**Figure 5:**
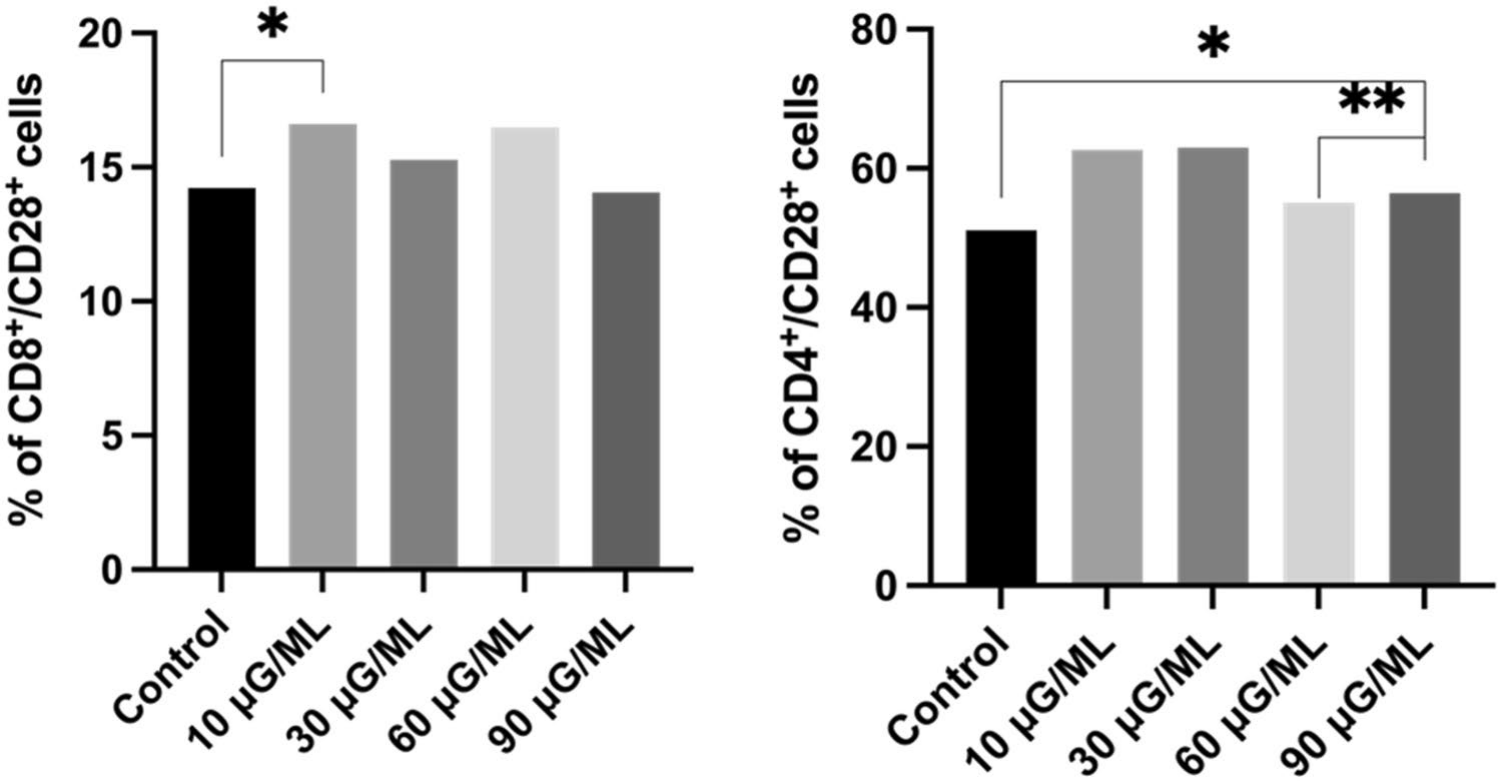
Representative bolt graph from the analysis of cell percentage with an expression of CD28 markers in a subpopulation of Tc and Th cells' *,**—describe statistically significant differences from Wilcoxon matched pair test, p-value < 0.05 (individual p-value for each group are described in Table 1).

In the CD8-positive T cell subpopulation, slightly higher percentages of cells expressing the CD28 marker were observed in all nivolumab-treated cultures than in nontreated control cultures. The highest data were observed for 10 μg/ml nivolumab. The percentage of T helper CD28-positive cells was nearly 4.5 times higher than that of T cytotoxic CD28-positive cells in all nivolumab-treated cultures. There was a statistically significant difference in CD4^+^/CD28^+^ cell percentage for probes treated with 60 μg/ml nivolumab in comparison to 90 μg/ml nivolumab (p = 0.0284). The representative graphs' analysis is shown in Fig. 5.

*5. Assessment of the percentage of Th and Tc cells with PD-1 (CD279) expression*

**Figure 6:**
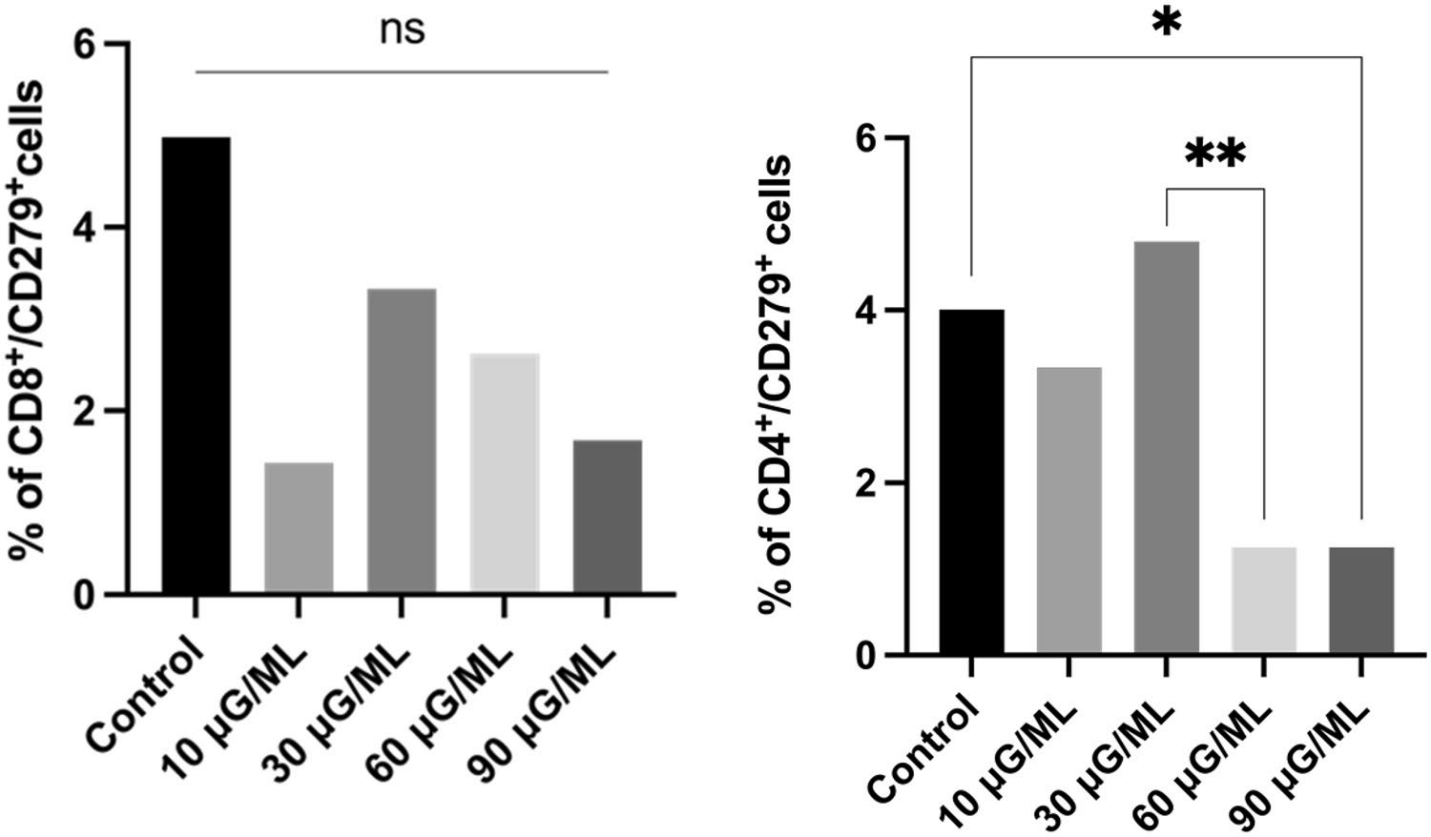
Representative bolt graph from the analysis of cell percentage with an expression of CD279 marker in a subpopulation of Tc and Th cells' *,**—describe statistically significant differences from Wilcoxon matched pair test, p-value < 0.05 (individual p-value for each group are described in Table 1), ns – nonsignificant.

A nonsignificant decrease in the percentage of PD-1-positive cells was observed in CD-8-positive cells after culture with different nivolumab concentrations compared with the control culture. However, significant changes in the percentage of PD-1-positive cells within CD-4-positive cells was observed only in the control compared to 90 μg/ml nivolumab and in the 30 μg/ml compared to 60 μg/ml nivolumab (p = 0.0277). The representative graph analysis is presented in Fig. 6.

## Discussion

In the present study, we analyzed the influence of different concentrations of anti-PD-1 antibodies on the vital function of T lymphocytes in an in vitro cell culture model. Nivolumab—an anti-PD-1 fully human monoclonal antibody has been widely used in clinical practice with high effectiveness; nonetheless, a relatively high percentage of adverse reactions have been reported. According to clinical data, PD-L1 expression on cancer cells does not constitute a critical and ideal prediction factor for PD-1/PD-L1 checkpoint blockade treatment in cancer patients. While blocking the PD-1/PD-L1 pathway, nivolumab should reverse the process of exhausting effector T lymphocytes and intensify T cell proliferation and lymphocyte elimination from the tumor microenvironment. Consequently, this action is intended to direct the dominant polarization of mature CD4-positive lymphocytes into CD8-positive cytotoxic T lymphocytes. The key step to achieving the therapeutic effect is restoring lymphocyte viability and cytotoxic capacity.

A brief regression should be added here. In clinical practice, routine diagnostic doesn’t include analysis of tumor immunological type. One of the reasons is the quality of biopsy samples. A significant impact in the immunosuppressive condition development and tumor sensitivity to anti-PD-1/PD-L1 therapy in cancer patients have myeloid-derived suppressor cells (MDSC). Chronic inflammation conditions related to cancer disease affect myelopoiesis and granulopoiesis. Hence, in cancer patient’s high subset of myeloid-derived suppressor cells is observed. In MDSC pull about 2/3 of cells belong to polymorphonuclear myeloid-derived suppressor cells (PMN-MDSC or g-MDSC) and 1/3 of cells are classified as monocytic myeloid-derived suppressors cells (m-MDSC)^7^. Among tumor-related factors involved in the generation of MDSCs and their recruitment from bone marrow to tumor microenvironment are mediated by granulocyte–macrophage colony-stimulating factor (GM-CSF), IL-6, indoleamine 2,3-dioxygenase (IDO) and chemokines (e.g. CCL26, CXCL12, CXCL8). Crucial role in the stimulation of MDSCs’ differentiation into highly immunosuppressive cell types play several factors released in TME: GM-CSF, IL-1b, IL-6, IL-10, IL-23, IFN-g, TNF-a, TGF-b and chemokines (e.g. CCL2, CCL3, CCL4, CCL5). A significant point for tumor immunity and the potential effectiveness of ICI’s therapy is a cross-talk between MDSC and regulatory T cells. T_reg_ activates immunosuppressive functions of MDSC through induction of B7-H1 (PD-L1), B7-H3, B7-H4, and IL-10 expression on MDCSs. Herein, MDSC released chemokines related to CCR5^+^ expression on T_reg_. One of the key mediators involved in M-MDSC transformation into pro-tumorigenic M2-macrophages and inhibition of effector T cells is a TGF-b. G-MDSC could be differentiated into tumor-associated neutrophils, which immunosuppressive functions are mediated by metabolites (e.g. Arg-1, ROS, NO) and soluble regulatory factors IL-10, MMP-9, and VEGF^7–9^. The factors described above are highly important in the context of tumor immunity and its sensitivity to ICI’s therapy as taking a certain role both in the formation of tumor infiltrated lymphocytes profile and antigen presentation cells activity. Nevertheless, the presence of MDSCs and soluble regulatory factors related to tumor immunity in peripheral blood samples is currently one of the important research subjects in cancer immunotherapy studies and should be validated. In this preliminary research, our aim was to take point on the ability of autologous T cells to be activated under ani-PD-1 treatment with simultaneous antigen presentation from dendritic cells. Of note, the ability of CD14 + cells to proliferate and activate autologous T cells is the significant to point about patients’ immune system condition. Both cytokines and chemokines released by tumor are involved in the immunosuppression process, which is manifests by malfunctioning of immune cells' phenotype and their presence in TME.

The analysis of chemokines and cytokines profile and their impact on patients’ response to immune therapy is significant but was not crucial in our research. In this work, we proposed simultaneous use of adoptive immune therapy with anti-PD-1 therapy.

The first part of our study presented a method concerning obtaining mature dendritic cells that are capable of antigen presentation and activation of T cell polarization without the use of bacterial lipopolysaccharide (LPS) or staphylococcal enterotoxin B (SEB). According to published original research, LPS or SEB induces IFN-γ dependent on upregulation of PD-L1 expression on tumor cells and immune cells infiltrating the tumor microenvironment^10^. As interferon plays a dual role in tumor immunology, this technique does not appear to be advisable for obtaining mature dendritic cells, especially in cancer patients^11^. In the present study, we used CD14-positive cells isolated from the peripheral blood of patients with advanced lung adenocarcinoma. The method used to generate mature dendritic cells is the standard technique employed in laboratory practice (6-day incubation with the addition of GM-CSF and IL-4). As a novelty, we used tumor oligopeptides typical of lung adenocarcinoma and TNF-α stimulation on the 6th day of incubation. Generated DCs express markers involved in antigen presentation (CD80/86, CD83, CD1a, CD209 molecules) and helper T cell proliferation activation (CD11c). They also expressed PD-L1/2 molecules, but the expression of B7-H4 was not reported. The B7-H4 molecule has strong inhibitory properties on the activation and polarization of mature T cells into effector T cells. The lack of B7-H4 expression on DCs made it possible to analyze the effectiveness of PD-1/PD-L1/2 trial blockade without a strong coinhibitory signal. The main difference in the generation of mature DCs in our work is an additional application of TNF-α and tumor oligopeptides MUC 1.1, MUC 1.2, MAGE A.3^10–12^. All oligopeptides were previously used for cancer vaccine production. MUC1 is a membrane protein typically expressed in lung cells and overexpressed with or without polarization in lung adenocarcinoma cells and is involved in cell-to-cell and cell-to-matrix interactions. MUC1 could also be involved in the exhaustion of T cell effector function in the tumor microenvironment^13–15^. MAGE-A3 protein is involved in the epithelial-mesenchymal transformation process and is linked to faster disease progression^16–19^. Third, a noncommonly used molecule for mature DC generation was TNF-α, which is involved in the upregulation of the NF-κB pathway and participates in tumor progression and chemo- and immunotherapy resistance in lung adenocarcinoma^20–22^. Based on previously published data and the results of our study, we suggest that the use of MUC1 and MAGE-A3 in combination with TNF-α is a more reliable approach to generate autologous dendritic cells for basic research on cancer immunology. The use of the mentioned tumor oligopeptides allowed the possibility of generating dendritic cells fully capable of forming a complete immune synapse and initiating immature T cell polarization into the Th1 subpopulation. It also allowed a more "natural" immune phenotype than when LPS or SEB were used. We proved that the generation of fully functional dendritic cells from lung cancer patients possibly indicates a lack of functional exhaustion of the immune system in the peripheral blood.

In the present study, we observed a significantly increased percentage of activated T cells after nivolumab treatment for all tested concentrations compared to the control probe. However, the results showed considerably increased data for 10 μg/ml nivolumab. CD25 and CD69 markers are typical for early activated T lymphocytes. The CD95 marker is involved in tumor apoptosis activation in CD8-positive lymphocytes. We observed increased T cells in the early activation stage after nivolumab treatment at all tested doses compared to the control. We also analyzed the expression of the CD28 molecule, as it is a key particle in immune synapse formation. The percentage of cells expressing CD28 after nivolumab treatment was not significantly higher than that of the control probe. However, it was nearly four times higher for CD4-positive cells than for CD8-positive cells. We also observed a significantly higher percentage of cells expressing receptors for interleukin-2 (CD25) and interleukin-4 (CD124) in CD4-positive cells after nivolumab treatment compared with the control. IL-2 is critical for T cells to polarize into cytotoxic T cells and activate natural killer cells^23^. Similarly, IL-4 is involved in activating NK cells, which play a leading role in tumor apoptosis activation^24^. The expression of PD-1 on CD4- and CD8-positive cells was lower after nivolumab treatment, but it was not significant when compared with the nontreated control. Of note, the nivolumab antibody could have a different extracellular PD-1 target point than the anti-PD-1 antibody used for flow cytometric analysis.

Only a few original papers describe in vitro studies with nivolumab's influence on cells from lung adenocarcinoma-bearing patients. There is also a lack of data regarding the proper nivolumab dose for in vitro research. There was a previously reported nivolumab dose for in vitro analysis, but the methodology and aim of that study differed from those of the present study. Vetrei et al. reported a 15 μg/ml dose of nivolumab for an in vitro study^25^. Wang Ch. et al. showed that nivolumab activates T cell proliferation even at a concentration of 1.5 ng/ml, but antigen presentation is required^12^. Selby et al. pointed to nivolumab's ability to stimulate CD8 + cytotoxic function and CD4 + effector function, but it does not affect the proliferation of naïve T cells^10^. Pharmacodynamic and pharmacokinetic analysis from the I phase trial, according to Centanni M. et al., for the ex vivo dose range > 0.1 μg/ml. They also confirmed no dose-related side effects in grade 3–5 tumors analyzed in 342 patients treated with 0.1–10 mg/kg nivolumab^26^. The most important relation observed in basic research is not always confirmed clinically. Drug dose is an important factor for therapy effectiveness and safety depending on its pharmacological properties. The major challenge for ICI therapy is reducing several side effects, including life-threatening conditions or death, during therapy.

Accordingly, to data from phase III clinical trials, the median time for the appearance of side effects after nivolumab treatment, such as hyperthyroid, pneumonitis, colitis, hyperthyroid, and hepatitis renal dysfunction, took place between the fourth and tenth weeks after therapy started^27^. Nevertheless, the more common adverse effects of ICI therapy in third or fourth grade are fatigue, dermatological changes, and gastrointestinal disorders. The intensity of the mentioned adverse effects often obliges discontinuing immunotherapy, which implicates a poor prognosis for patients. Analysis of antidrug antibody presence in patients with solid tumors from 6 clinical trials during therapy showed no clinical significance of nivolumab immunogenicity^28^. Wang X. et al. described that the nivolumab time-averaged concentration after the first dose was not significantly related to objective response and overall survival in advanced-stage melanoma cases. They also confirmed a total of 3 mg/kg nivolumab once every two weeks (Q2 W) for solid tumor treatment, including NSCLC^29^. Long G. et al. also confirmed no differences in treatment benefits and safety between lower doses (480 mg/kg every week) vs. 3 mg/kg every two weeks^30^. Desnoyer A. et al. revealed an association between changes in drug clearance and the effectiveness of nivolumab treatment, but there were no clinically significant factors involved. The authors also confirmed no-dose relation for nivolumab treatment^31^. A few retrospective multicenter studies showed no significant differences in progression-free survival (PFS) times beyond standard nivolumab treatment with 3 mg/kg nivolumab once every three-eight weeks and low-dose 240 mg/kg nivolumab every two weeks; 480 mg/kg nivolumab every four weeks; and 0.1 mg/kg or 100 mg/kg nivolumab every three weeks during treatment in patients with advanced NSCLC. Third phase clinical trials CheckMate-066, -025, -057, and -017 showed similar PFS times and frequencies of side effects of 480 mg/kg Q4 W nivolumab dose compared to the standard scheme in patients after disease progression. Additionally, preliminary data from CheckMate-384 in pretreated patients with advanced-stage IIIB/IV NSCLC showed no significant differences between subgroups treated with 480 mg/kg Q4 W vs. 240 mg/kg Q2 W. The recorded adverse effect percentage was similar, with data from patients treated with 3 mg/kg Q3 W^32^. The more frequent use of lower doses might be more convenient for patients during the pandemic, according to cited research.

The key for improving immunotherapy's efficiency by lowering side effects is to achieve the optimal T cells activation level. Nowadays, it is more important due to possibility of subcutaneous anti-PD-1 drugs administration. Johnson M et al. revealed the feasibility of monthly subcutaneous administration of the anti-PD-1 antibody. An intravenous dose escalation (0.5, 1, 3, or 10 mg/kg) of anti-PD-1 antibody was administered every 3 weeks, or a subcutaneous dose of 300 mg was administered every 4 weeks. The authors did not observe dose-limiting toxic effects, while grade 3 or higher treatment-related adverse events occurred in 4 (16%) patients treated intravenously and in 1 (6.7%) patient treated subcutaneously. Thus, monthly subcutaneous administration of anti-PD-1 offers a convenient and effective alternative to currently available intravenously administered checkpoint inhibitors^33^.

The PD-L1 expression level is the only basic predictive marker in clinical practice for immune checkpoint inhibitor therapies. Accordingly, in European registration, no PD-L1 expression is required for nivolumab treatment, as data from various clinical trials have shown clinical benefit even in patients with PD-L1 expression levels < 1%^34^. The effectiveness of nivolumab in first-line monotherapy was lower than that for chemotherapy. Hence, the recommendations advise the use of nivolumab in second-line cancer therapy. Another important point is combined immunotherapy. Significantly longer PFS was observed for patients treated with nivolumab in combination with ipilimumab as a first-line treatment. The percentage of side effects in patients treated with this combination was slightly lower than that after chemotherapy^35^. The Food and Drug Agency approved using nivolumab in combination with ipilimumab for first-line treatment in two dose-time combination strategies. The first involves 3 mg/kg nivolumab every two weeks and ipilimumab at a dose of 1 mg/kg every six weeks administered intravenously. The second is 360 mg/kg nivolumab every three weeks and 1 mg/kg ipilimumab every six weeks and two weeks of platinum-doublet chemotherapy with the same type of administration. Accordingly, to the European Medicine Agency, the nivolumab dose for advanced NSCLC treatment should be 240 mg/kg distributed intravenously every two weeks in monotherapy and 360 mg/kg every three weeks in combination with 1 mg/kg ipilimumab every six weeks and platinum-based chemotherapy every three weeks. Despite the higher effectiveness of immunotherapy in lung cancer treatment, several specific side effects indicate the need for optimizing the drug dose^36^. The failure of dependence in drug-response correlation for nivolumab treatment suggests a necessity to improve the methods of nivolumab use in clinical practice, e.g., in combination with other ICIs or modification of drug administration methods^10,28,35,37,38^. Seldom-distributed higher doses of nivolumab might be more convenient for cancer-bearing patients, especially during a pandemic of SARS Covid-19. It is crucial to limit patients' hospitalization time without impacting their treatment program.

There is lake of date about pharmacokinetics and pharmacodynamics of nivolumab. This kind of information is critical to analyse mechanism underlying nivolumab therapy and its related effects. For example, the concentration of the drug that goes directly to the tumor and which reaches the metastatic niche is unknown.

In conclusion, the possibility of generating autologous mature dendritic cells capable of activating Th cells from PBMCs of patients in stage IIIB/IV lung adenocarcinoma allows us to conclude that the patient's immunology system was not exhausted. Furthermore, data from clinical trials did not show a clear relationship between the occurrence and intensity of adverse effects and the administered dose of nivolumab. Therefore, we could conclude that the possibility of adverse effect development after immunotherapy mainly depends on the patients' immune system condition.

The future perspective regarding the effectiveness of immunotherapy should be directed at minimizing the side effects while maintaining the stimulation of T lymphocyte activity. However, this requires *in vit*ro tests, which should always be the basis for the development of new immunotherapy combinations and their administration.

## Materials and methods

This study was approved by Bioethical Committee at the Medical University of Lublin. Consent ID number: KE-0254/318/2018. Informed consent was obtained from all subjects and/or their legal guardian(s). All experiments were performed in accordance with relevant guidelines and regulations.

### Characteristic of the study group

Peripheral blood samples were obtained from patients with lung adenocarcinoma during a routine diagnostic process in the Department of Pneumonology, Oncology and Allergology, Medical University of Lublin (Poland). Criteria for patient's inclusion were following:

1. Locally advanced nonoperative non-small-cell lung cancer in the III or IV stage of advancement before initiating the treatment. The stage of lung cancer advancement was determined mainly based on CT taken no later than two weeks prior to the commencement of the study.
2. Patients who did not receive growth factors or immune-stimulating factors (e.g., erythropoietin or granulocyte macrophage-colony growth factor for treating anemia or granulocytopenia) six months prior to the commencement of the study.
3. Patients without confirmed another cancer type or without cancer symptoms during the last five years of observation.
4. Patients without immune defense disease or nontreated with immunodeficient drugs.
5. Patients without "driver" mutations in *EGFR* or *BRAF* genes or rearrangements in *ROS1* or *ALK* genes.

All analyses were based on data retrieved from peripheral blood samples collected from patients prior to the commencement of the study.

### Isolation of autologous mononuclear cells

Peripheral blood mononuclear cells (PBMCs) were isolated from venue blood collected into a probe with heparin less than an hour after collection. Cell separation was performed by density gradient centrifugation with 3 ml of Lymphoprep™ (Stemcell) per 10 ml of blood diluted in PBS (Mg^2+^/Ca^2+^ free) in a 1:1 ratio. Next, we collected the CD14^+^ cell fraction and T lymphocyte fraction from isolated PBMCs during separation in a magnetic field. The whole procedure was performed according to the manufacturer’s protocol. For CD14^+^ isolation, CD14 microbeads (MACS) and MACS buffer (2 mM EDTA, 0.5% FBS) were used. Isolated and purified CD14-positive cells were used to establish dendritic cell cultures. The T cell pellet was suspended in 1 ml of CryoMaxx SF (PAA Cell Culture Company) and stored at -80 °C for 6 days.

### Generation of mature dendritic cells

A purified CD14-positive cell pellet was suspended in GMP Dendritic Cell Medium (Cell Genix) and transferred to a culture bottle. Next, the cell culture medium was supplemented with IL-4 (500 IU/ml), GM-CSF (1000 IU/ml) and antibiotic mix (10^4^ U penicillin, 10 mg/ml streptomycin, 25 μg/ml amphotericin) (Sigma-Aldrich). The cultures were supplemented with IL-4 and GM-CSF every 48 h. After 120 h of culture, TNF-α (50 ng/ml) and oncogene peptides MUC1.1, MUC1.1.2, and MAGE A.3 (Pepscan, Belgium) at 50 ng/ml concentration per peptide were added to the cell culture to stimulate dendritic cell maturation. After 24 h of incubation, the immunophenotype of dendritic cells was checked by flow cytometry, and mixed cocultures were prepared.

### Mixed coculture of lymphocytes and mature dendritic cells with different nivolumab concentrations

T cells fraction was thawed and washed with PBS. The cell pellet was resuspended in MACS buffer and incubated with antibody T cell mix. After incubation, a pure fraction of T cells was isolated in a magnetic field according to the manufacturer’s protocol from MACS and resuspended in DC medium. T cell mixed culture with generated mature DCs was performed on a 6-well plate. The ratio of T lymphocytes to mature dendritic cells was 10:1. Nivolumab concentrations were calculated based on pharmacokinetics information included in the product safety data sheet. The chosen concentration range includes average and extreme drug concentration values that may enter tumors in patients' bodies. The following nivolumab concentrations were added to the mixed culture: 10 μg/ml, 30 μg/ml, 60 μg/ml, and 90 μg/ml. The mixed cultures were conducted for 48 h, and after that, flow cytometry analysis of the cell phenotype was performed. The culture with lymphocytes and dendritic cells without nivolumab addition served as a control culture (Table 1).

**Table 1:** Median values for cell percentage expressed analyzed markers with p value from Wilcoxon matched pair test (*—p < 0.05; **—p < 0.01; ***—p < 0.005).

	Control (%)	10 μg/ml % of cells (*p* for control vs. 10 μg/ml)	30 μg/ml % of cells, (*p* for control vs. 10 μg/ml)	60 μg/ml % of cells, (*p* for control vs. 10 μg/ml)	90 μg/ml % of cells, (*p* for control vs. 10 μg/ml)
CD8^+^/CD25^+^	6.44	13.23 (*0.016)	10.75 (ns)	11.96 (*0.046)	11.7 (*0.0284)
CD8^+^/CD69^+^	3.94	10.6 (ns)	7.57 (ns)	9.78 ns	8.18 ns
CD8^+^/CD95^+^	31.9	37.64 (ns)	35.66 ns	34.08 ns	36.09 ns
CD4^+^/CD25^+^	41.2	65.04 (**0.008)	33.38 (ns)	58.01 (ns)	51.92 (ns)
CD4^+^/CD69^+^	15.51	33.33 (ns)	10.17 (ns)	25.45 (ns)	18.78 (ns)
CD4^+^/CD95^+^	35.45	42.17 (ns)	40.39 (ns)	41.33 (ns)	43.89 (ns)
CD8^+^/CD107a^+^	1.37	9.69 (***0.004)	5.93 (***0.003)	8.64 (***0.005)	7.685 (**0.007)
CD4^+^/CD107a^+^	0.34	1.68 (ns)	2.06 (*0.046)	2.61 (*0.028)	2.19 (*0.046)
CD4^+^/CD124^+^	18.93	53.54 (*0.033)	34.69 (ns)	45.76 (ns)	39.87 (ns)
CD4^+^/CD212^+^	14.01	29.54 (ns)	9.65 ns	21.67 (ns_	20.78 ns
CD8^+^/CD28^+^	12.59	18.65 (*0.026)	14.56 (ns)	18.32 (ns)	17.62 (ns)
CD4^+^/CD28^+^	65.95	76.15 (ns)	74.39 (ns)	76.38 (ns)	76.23 (*0.0468)
CD8^+^/CD279^+^	16.18	3.69 (ns)	5.59 (ns)	4.88 (ns)	2.68 (ns)
CD4^+^/CD279^+^	8.84	6.93 (ns)	10.68 (ns)	2.98 (ns)	2.45 (*0.0464)

### Flow cytometry immunophenotyping of mature dendritic cells and lymphocytes after mixed cultures

After 48 h of culture incubation with different concentrations of nivolumab, the cells were harvested and washed with PBS. Next, the cells were incubated with fluorescently labeled antibodies (BD Bioscience) for cytometric analysis. The whole procedure was performed according to the manufacturer’s protocol. For intracellular marker analysis, permeabilization of the cell membrane was performed using a transcription factor buffer set (BD Bioscience). Cytometric analysis panels for CD4-positive and CD8-positive lymphocyte subpopulations as well as for the immunophenotype of generated dendritic cells are presented in Tables 2 and 3, respectively.

**Table 2:** Short characteristic of markers for T lymphocyte immunophenotyping.

**Activation markers**	
CD25	Analysis of T helper (CD4 +) and T cytotoxic (CD8 +) cells with expression of the interleukin's 2 receptor
CD69	Analysis of the early activation stage in T helper and T cytotoxic cells
CD95	Analysis of the early apoptotic stage in T helper and T cytotoxic cells
**Intracellular marker**	
107a	CD107a is a marker of degranulation on T cytotoxic cells and NK cells. CD8^+^CD107a^+^ cells could initiate the cytolysis of target cells
**Interleukin’s receptor**	
CD124 (IL-4R)	Analysis of the T helper polarization into Th1 subpopulation
CD212 (IL-12R)	Analysis of the T helper polarization into Th2 subpopulation
**Immune synapse receptor expression**	
CD28	Major costimulation marker, its expression is obligatory for the formation of an immune synapse
**Immune checkpoint inhibitor expression**	
CD279 (PD-1)	Analysis of T helper and T cytotoxic cells with PD-1 expression

**Table 3:** Short characteristics of markers for dendritic cell immunophenotyping.

**Immature monocyte**	
CD14	Analysis of the immature not differentiated monocytes
**Mature dendritic cells**	
CD1a	Analysis of DCs capable of presenting the antigens from cancer cells
CD11c	Analysis of DCs capable of inducing T helper polarization into Th2 subpopulation
CD80/86	Analysis of DCs capable of the formation of the immune synapse
CD83	
**DCs with the ability to activate T cells**	
CD209	Analysis of DCs' ability to activate T cells through cancer antigen presentation
**DCs with the ability to suppress T cells**	
B7 DC (PD-L2)	DCs positive for this marker express the ability to inhibit T cell function through the PD-1 pathway
CD274 (B7H1, PD-L1)	
B7 H4	DCs positive for this marker express the ability to inhibit T cell proliferation and anticancer activation

### Statistical analysis

Statistical analysis of the obtained results was based on the nonparametric Wilcoxon test (GraphPad Prism 9), comparing two related variables. A value of p < 0.05 (*) was considered statistically significant.
